# Progression of Alport Kidney Disease in *Col4a3* Knock Out Mice Is Independent of Sex or Macrophage Depletion by Clodronate Treatment

**DOI:** 10.1371/journal.pone.0141231

**Published:** 2015-11-10

**Authors:** Munkyung Kim, Alessandro Piaia, Neeta Shenoy, David Kagan, Berangere Gapp, Benjamin Kueng, Delphine Weber, William Dietrich, Iwona Ksiazek

**Affiliations:** 1 Developmental and Molecular Pathways, Novartis Institute for Biomedical Research, Basel, Switzerland; 2 Preclinical Safety, Novartis Institute for Biomedical Research, Basel, Switzerland; 3 Preclinical Safety, Novartis Institute for Biomedical Research, Cambridge, Massachusetts, Unites States of America; 4 Developmental and Molecular Pathways, Novartis Institute for Biomedical Research, Cambridge, Massachusetts, Unites States of America; UCL Institute of Child Health, UNITED KINGDOM

## Abstract

Alport syndrome is a genetic disease of collagen IV (α3, 4, 5) resulting in renal failure. This study was designed to investigate sex-phenotype correlations and evaluate the contribution of macrophage infiltration to disease progression using *Col4a3* knock out (Col4a3KO) mice, an established genetic model of autosomal recessive Alport syndrome. No sex differences in the evolution of body mass loss, renal pathology, biomarkers of tubular damage KIM-1 and NGAL, or deterioration of kidney function were observed during the life span of Col4a3KO mice. These findings confirm that, similar to human autosomal recessive Alport syndrome, female and male Col4a3KO mice develop renal failure at the same age and with similar severity. The specific contribution of macrophage infiltration to Alport disease, one of the prominent features of the disease in human and Col4a3KO mice, remains unknown. This study shows that depletion of kidney macrophages in Col4a3KO male mice by administration of clodronate liposomes, prior to clinical onset of disease and throughout the study period, does not protect the mice from renal failure and interstitial fibrosis, nor delay disease progression. These results suggest that therapy targeting macrophage recruitment to kidney is unlikely to be effective as treatment of Alport syndrome.

## Introduction

Alport syndrome is an inherited genetic disease which affects approximately 1 in 5000 people and is caused by mutations in the type IV collagen genes [1]. In particular, mutations in the type IV collagen α5 chain gene (*COL4A5*) are responsible for the X-linked form of the disease, which accounts for ~85% of the patients and mutations in the type IV collagen α3 or α4 chain gene (*COL4A3* or *COL4A4*) lead to the autosomal form of the Alport syndrome [2]. Type IV collagen assembles primarily as α3α4α5 heterotrimers in the adult glomerular basement membrane (GBM) and is one of the main structural components essential for GBM integrity and function. Mutations in any of the three collagen chains can result in defective assembly of the GBM leading to the renal pathology of Alport syndrome manifested by irregular thickening and splitting of the GBM, podocyte effacement, glomerulosclerosis with extracellular matrix deposition, kidney fibrosis, and ultimately, end stage renal disease (ESRD) early in life [3,4].

*Col4a3*-deficient (Col4a3KO) mice, one of murine models of Alport syndrome [5–8], are developed by gene targeting at the *Col4A3* locus and raised on a 129/SvJ genetic background [6]. In the absence of type IV collagen α3, α4, and α5 chains, mice develop progressive glomerulonephritis as well as ESRD and die at an age of approximately 10 weeks [6,9]. The structural and functional manifestation of renal pathology of Col4a3KO mice closely resembles that of human Alport syndrome, making Col4a3KO mice an ideal model to understand Alport pathology. The translatability of Col4a3KO model for the autosomal recessive form of Alport syndrome is demonstrated by animal studies with Col4a3KO mice that have successfully assisted in identifying effective therapies for Alport patients. Well-established evidence comes from RAAS blockage with ACE inhibitors which delays progression to renal replacement therapies in humans with Alport syndrome [10,11] and is effective in delaying renal failure in Col4a3KO mice [9]. While i) human autosomal form of Alport syndrome is shown to affect males and females equally [12], and ii) mice bearing Col4a4 splice site mutation, another model of Alport syndrome, show similar progression of albuminuria in males and females [5], relatively little is known about sex-specific susceptibility to disease progression in Col4a3KO mice. Especially, sex of the mice was not specified in the previously published studies which Col4a3KO mice [6,7,13]. One of the goals of this study was to determine whether sex has a significant impact on the onset and progression of kidney disease in Col4a3KO mice.

It is well established that interstitial inflammation is a prominent feature of progressive renal diseases including, Alport syndrome. As early as 1961, Whalen and colleagues reported the presence of CD68-positive foam cells in human Alport syndrome [14]. Foam cells belong to the monocyte-macrophage lineage and acquire their ‘foamy’ appearance owing to accumulation of fat. Extensive macrophage infiltration is also reported for the Col4a3KO kidney with a strong correlation to the severity of kidney injury and fibrosis [15,16]. In spite of the association of macrophages with Alport syndrome, the contribution of macrophage infiltration to the progression of Alport syndrome remains elusive. Previous studies in Col4a3KO mice with agents attenuating monocyte-macrophage recruitment to kidney have yielded equivocal results, with one study showing improved renal pathology and mice survival [17] and another showing no improvement [18].

Clodronate is a transient, selective, and systemically acting macrophage-depleting agent [19,20]. The phagocytosis-mediated uptake of clodronate leads to suicidal apoptosis and abrogation of macrophage functions in the targeted organs. This depletion strategy has been successfully applied to ablate macrophages in other animal models of acute and chronic renal diseases [21,22], but has not yet been reported in Alport syndrome mice.

This study was conducted to investigate i) the effect of macrophage depletion in the progression of Alport disease in Col4a3KO mice and ii) any sex-specific susceptibility of these mice to Alport disease. Animal weights, renal pathology, and renal biomarkers of function and injury were used to assess disease progression over time.

## Materials and Methods

### Mice

Col4a3KO mice with 129/SvJ background (129-Col4a3^tm1Dec^/J) were purchased from The Jackson Laboratory (Bar Harbor, ME, USA) and maintained as a heterozygous colony. All animal studies were performed in accordance with the Swiss Guidelines for animal experimentation and approved by the animal care committee of the Canton Basel-Stadt, Switzerland on 25 Nov 2011 (License number: BS-2409).

### Animal studies

The sex and genotype of Col4a3KO mice was determined at 3 to 4 weeks of age. Both sexes of Col4a3KO mice and their wild-type littermate controls of the same sex were used for the experiments unless stated differently. To analyze the sex effect, the study was initiated in 4 weeks old mice and terminated when the mice reached the age of 9–10 weeks or lost more than 20% of their body weight. The study with clodronate liposomes (CL) or PBS liposomes (PBSL) (Encapsula NanoSciences, Brentwood, TN, USA) was initiated in 4 week old mice and terminated when the mice reached the age of 8 weeks. Liposomes (200 μl/mouse) were injected intraperitoneally for 2 consecutive days, followed by every second day of administration until end of the study.

### Urine and blood analysis

Urine was sampled weekly and analyzed for albumin, creatinine, NGAL, and KIM-1. Albumin was measured using the Albuwell M kit (Exocell, Philadelphia, PA, USA) and normalized to creatinine levels analyzed by Aution urine analysis system (Arkray, Kyoto, Japan).

Mouse KIM-1 was analyzed with an immunoassay using an anti-mouse rat monoclonal and an anti-mouse goat polyclonal as the capture and detection reagent, respectively (R&D Systems, Minneapolis, MN, USA) on an SI6000 from Mesoscale Discovery (MSD, Rockville, MD, USA). 30 μL of capture antibody (4 μg/mL in PBS) was incubated overnight on MSD standard plates at 4°C. The plate was washed 3X with PBS followed by the addition of 25 μl of urine (1:4 dilution in MSD diluent 5) and incubated for 1h at room temp. Incubation with the secondary antibody for 1h was followed by a wash and application of MSD Sulfo-Tagged anti-goat antibody for 1h. After another wash, 150 μl of MSD Read Buffer T was added, and the plates read on an MSD SI 6000. Data were analyzed on MSD Discovery Workbench software. NGAL and Albumin were assayed at 1:1000 and 1:100 dilutions, respectively as per manufacturer’s instruction using kits from Bioporto (Hellerup, Denmark) and Abnova (Taiwan, Republic of China), respectively. KIM-1 and NGAL were normalized to creatinine analyzed using the Urinary Detection Kit from Arbor Assays (Ann Arbor, MI, USA) at a 1:25 dilution in H_2_O and assayed as per manufacturer’s instructions. Plates were read on a SpectraMax M5 from Molecular Devices (Sunnyvale, CA, USA), analyzed with SoftMax Pro v5.

Blood was sampled every two weeks from V. sublingualis into Microtainer tubes (BD biosciences, San Jose, CA, USA) under isoflurane anesthesia. Serum was used to measure blood urea nitrogen by Spotchem EZ Automated analyzer and Spotchem ll reagent strip (Arkray).

### Immunohistochemistry and histology

Kidneys were fixed in 10% buffered formalin for 48h at room temp and processed for embedding in paraffin using standard procedures. Immunohistochemical staining for F4/80 was performed using an automated Ventana Discovery XT Platform (Ventana medical systems, Tucson, AZ, USA). Sections, pretreated with protease (Ventana), were incubated with Peroxidazed 1 (Biocare medical, Concord, CA, USA) and stained with monoclonal rat anti-mouse F4/80 antibodies (1:100; #MCA497G, ABD Serotec, Oxford, UK) for 48 min. Reaction was detected with the OmniMap anti-Rt HRP (Ventana) and ChromoMap DAB Kit (Ventana), followed by counterstaining with hematoxylin. For α-SMA staining, sections were treated with 0.5% H_2_O_2_ in methanol for 20 min, followed by 20 min incubation with monoclonal mouse anti-human α-SMA antibodies (1:25; #M0851, DAKO, Glostrup, Denmark), detection by ARK™ Peroxidase kit (DAKO), and counterstaining with hematoxylin. For Sirius red staining, Picrosirius Red solution and 0.04% Light Green solution (EMS, Hatfield, PA, USA) were used according to the manufacturer’s recommendations. Sections were stained with hematoxylin and eosin (H&E) and Periodic acid-Schiff (PAS) using standard protocols. Digital images were obtained with a ScanScope XT system (Leica, Nussloch, Germany). For Immunofluorescence staining, sections were incubated overnight at 4°C with monoclonal rat-anti mouse F4/80 antibodies (1:20; #MCA497G, ABD Serotec, Oxford, UK) and polyclonal goat-anti mouse CD206 antibodies (1:100; #AF2535, R&D systems, Minneapolis, MN, USA) followed by 1h incubation with donkey anti-rat IgG Alexa fluor 549 (1:500, Jackson Immunoresearch, Westgrove, PA, USA) and donkey anti-goat IgG Alexa fluor 488 (1:500, Life technologies, Gaithersburg, MD, USA) and counterstaining with hematoxylin. Digital images were obtained with LSM700 confocal microscope (Carl Zeiss Microscopy, Gottingen, Germany).

### Quantification of immunohistochemical staining

For the quantification of Sirius red and chromogenic staining, area %, defined as stained area per total surface area of entire kidney including cortex and medulla, was obtained with Image Scope software (Leica) using the Positive Pixel Count algorithm. For the analysis of F4/80/CD206 staining, five representative images from kidneys sections were analyzed by ImageJ (NIH, Bethesda, MD, USA). Colocalization Threshold plugin of ImageJ was used to calculate the F4/80^+^/CD206^+^ area. F4/80^+^/CD206^-^ was calculated as a difference between F4/80^+^ and F4/80^+^/CD206^+^ area. The data were presented as Area %.

### Histopathological evaluation

Glomerular sclerosis percentages were assessed by counting the number of segmental to global sclerotic glomeruli, and other glomeruli with variable changes but with patent vessels on H&E sections. Tubulointerstitial change indices were obtained from H&E slides as the mean value between the semiquantitive score assigned to each change (namely tubular degeneration/atrophy, tubular dilation, tubular casts, and interstitial fibrosis, with four-grade score system related to the extent of the change: 0, no lesion; 1, < 25% affected tubuli; 2, 25 to 50% affected tubuli; 3, 51 to 75% of affected tubuli; 4, > 75% affected tubuli).

### Real-time qRT-PCR analysis

Kidney tissue was snap frozen in liquid nitrogen and homogenized with the FastPrep-24 (MP Biomedicals, Santa Ana, CA, USA) system. RNA was isolated using an RNeasy purification kit (Qiagen, Venlo, Netherlands) according to the manufacturer’s recommendations. Quantitative real-time PCR was performed using predesigned probes (Table 1), Taqman Universal Master Mix and the ABI Prism 7900 HT Sequence Detection System (Applied Biosystems, Foster City, CA, USA). Gene expression was normalized to 18S rRNA expression.

**Table 1:** Probes used for quantitative real-time PCR analysis.

Gene	Catalog number
Col3a1	Mm00802331_m1
F4/80	Mm00802529_m1
Thy1	Mm00493681_m1
TGFβ	Mm00441724_m1
Fibronectin	Mm01256744_m1
PAI-1	Mm00435860_m1
MMP-9	Mm00600163_m1
TIMP-1	Mm00441818_m1
TNFα	Mm00443258_m1
IP-10	Mm00445235_m1
MCP-1	Mm99999056_m1
CD204	Mm00446214_m1
CD206	Mm00485148_m1
18S rRNA	4319413E

### Statistical analysis

The results are presented as mean± SEM. Unpaired t test was used for the comparisons between two groups while Welch corrected ANOVA followed by posthoc pairwise t-test with a Bonferroni correction was used to evaluate differences between multiple groups. * or #: p< 0.05; ** or ##: p <0.01; *** or ###: p <0.001. For repeated measurements, data were analyzed using mixed model data analysis followed by posthoc Fisher’s LSD test.

## Results

### Body weight in Col4a3KO mice of both sexes

**Fig 1:**
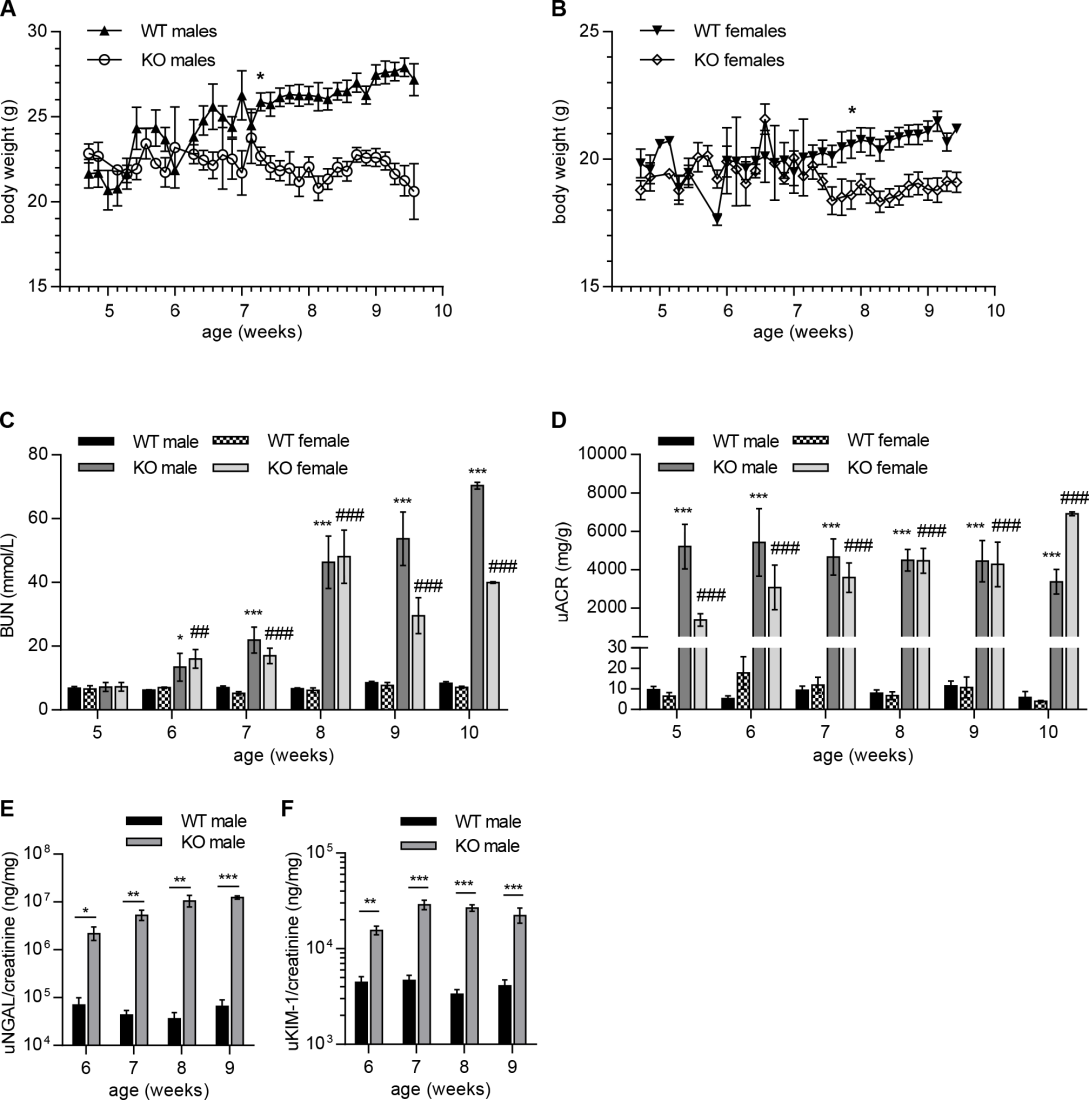
Evolution of body weight and renal function in Col4a3KO and WT mice. (**A, B**) Reduction of body weights in male (**A**) and female (**B**) KO mice as compared to WT littermates at 7 weeks 2 days and 7 weeks 6 days of age, respectively. (**C-F**) Increase in renal dysfunction biomarkers (**C, D**) accompanied an increase in kidney injury biomarkers (**E, F**) in KO mice after 5 weeks of age. Serum BUN (**C**), urinary ACR (**D**), urinary NGAL (**E**), and urinary KIM-1 (**F**) levels were significantly increased in KO mice at 5–6 weeks of age and continued to increase until the end of the monitored period (10 weeks). Mixed model data analysis was used for the comparison between WT and KO mice of same sexes. *, significance vs WT males; #, significance vs WT females. (A, B) n = 10 to 15 mice per group. (C, D) n = 5 to 10 mice per group. (E, F) n = 3 to 5 mice per group.

Uremic cachexia is prevalent in chronic kidney disease including Alport syndrome and is associated with mortality [23,24]. To study the progression of cachexia in male and female Col4a3KO (KO) mice, change in body weights are reported in comparison to wild-type (WT) littermates of the same sex. Up to the first 7 weeks of life, the weights of KO mice were largely indistinguishable from their respective WT littermates (Fig 1A and 1B). After approximately 7 weeks of age, both male and female KO mice stopped gaining weight in contrast to their WT siblings, which continued to exhibit gradual increases in body weight. At the age of 7 weeks 2 days and 7 weeks 6 days (51 and 55 days), respectively, the male and female KO mice demonstrated significant lower body weights as compared to the age- and sex-matched WT siblings (12% and 10%, respectively). By 9 weeks of age, the male and female KO mice were lighter than age- and sex-matched WT littermates by approximately 18% and 11%, respectively.

### Change in renal function in Col4a3KO mice of both sexes

To assess whether KO mice exhibit sex-dependent alterations in the onset and the kinetics of renal function decline, serum urea nitrogen (BUN) and urinary albumin were monitored weekly for both sexes from the age of 5 to 10 weeks. As shown in Fig 1C, serum BUN levels in 5-weeks old KO mice were comparable to the WT littermates with no discernable differences between the sexes. However, by 6 weeks of age, both male and female KO displayed significantly elevated BUN levels as compared to their WT littermates (~2-fold). BUN levels gradually increased in both sexes of KO mice until 8 weeks of age; at which time a ca. 7-fold increase in BUN was measured. By 10 weeks of age, BUN levels were on average 9- and 6-fold higher in KO males and females, respectively, compared to their WT littermates. Levels of BUN were comparable for female and male KO mice at all measured time points, except for weeks 9 and 10. Urinary albumin/creatinine ratios (henceforth referred to as ACR) were assessed in KO males and females between 5 to 10 weeks of age (Fig 1D). ACR increased significantly in 5-week old KO mice with approximately 540- and 210-fold increases in males and females, respectively, compared to WT littermates. ACR remained elevated over the entire observation period in KO, with ca. 580-fold and 1700-fold increases in males and females, respectively over WT level by 10 weeks of age. ACR were comparable for female and male KO mice at all measured time points, except for weeks 5 and 10.

### Change in kidney injury biomarkers in Col4a3KO mice

To investigate kidney injury, levels of neutrophil gelatinase-associated lipocalin (NGAL) and kidney injury molecule-1 (KIM-1) were systematically measured and normalized to urinary creatinine in KO mice from 6–10 weeks of age. NGAL and KIM-1 levels showed marked increases in the week 6 urine samples as compared to the age-matched WT mice and these continued to increase gradually throughout the study period in both male (Fig 1E and 1F) and female mice (data not shown). Approximately 30- and 140-fold increases in NGAL were measured in 6-and 9-week old KO males, respectively over the WT level. KIM-1 levels were increased 3- and 5-fold over WT in 6-and 9-week old KO males, respectively. No sex differences in NGAL and KIM-1 levels were observed (data not shown).

### Renal pathology in male and female Col4a3KO mice

**Fig 2:**
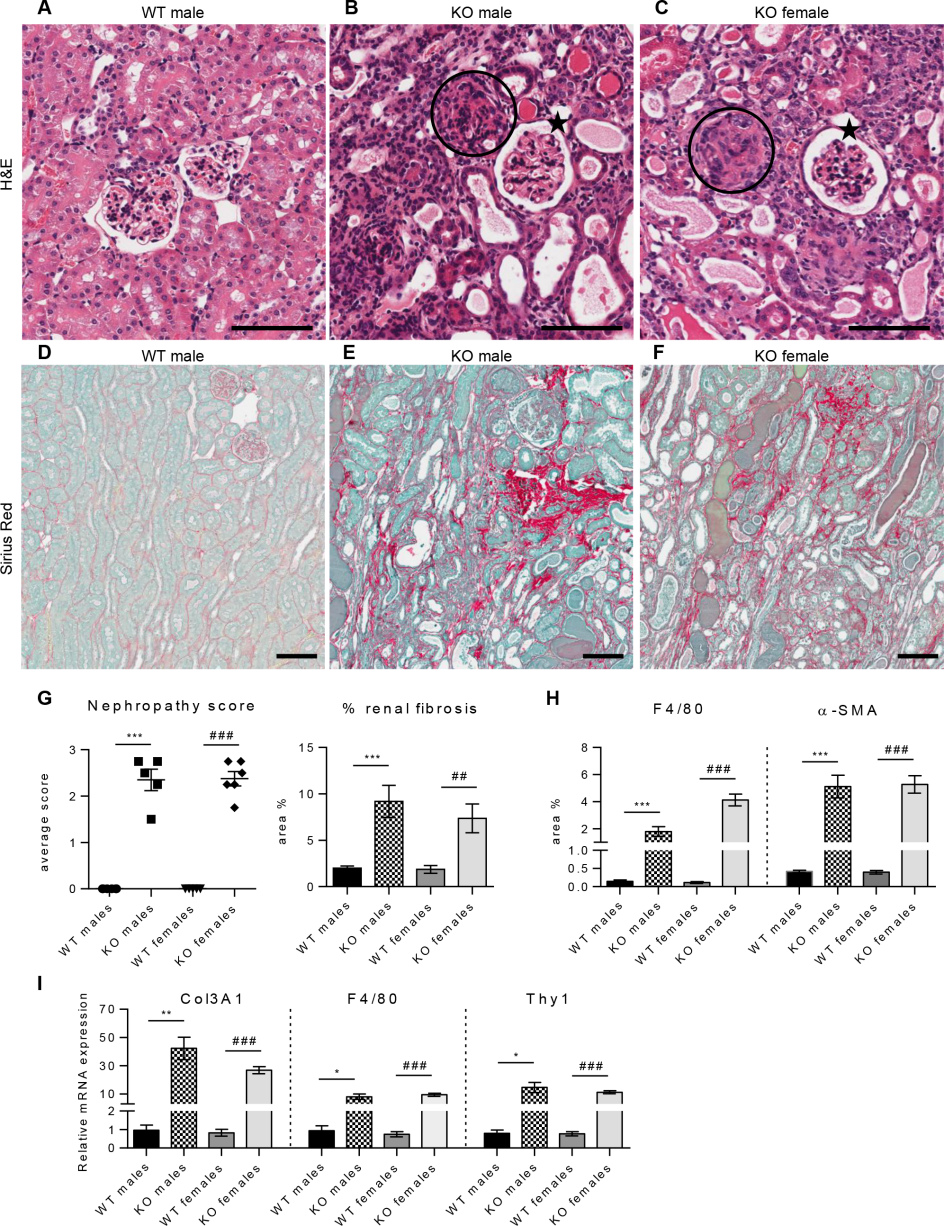
No differences in renal pathology between male and female Col4a3KO kidney at 8 weeks of age. (**A-C**) H&E-stained cortex from WT male (**A**), KO male (**B**), and KO female mouse (**C**). KO males and females showed glomerulosclerosis (circled) to varying degrees, with some glomeruli appearing less affected and almost normal (stars). Tubular changes were characterized by tubular dilatation and tubular atrophy. (**D-F**) Sirius red-stained cortex from WT male (**D**), KO male (**E**), and KO female mouse (**F**). WT showed a thin delineation of Sirius Red-stained fibers around tubuli, in KO this was variably thickened, especially in the transition zones between outer and inner stripes of the outer medulla and between outer medulla and cortex. (**G**) Nephropathy score assessed in H&E- and PAS-stained sections and quantitative analysis of Sirius Red positive interstitial fibrosis demonstrated significantly increased nephropathy and interstitial fibrosis in KO mice compared to WT littermates of the same sex with no differences between female and male mice. (**H**) Morphometric analysis of F4/80-positive macrophages and α-SMA-positive myofibroblasts showed significant increase of macrophages infiltrates and myofibroblasts deposition in both male and female KO kidney, compared to WT littermate controls of the same sex. The degree of changes was comparable in animals of both sexes with the exception of the F4/80-positive macrophage area being about two-fold higher in female than in male KO mice. (**I**) Real-time PCR analysis of Col3A1, F4/80 and Thy-1 mRNA showed marked upregulation in the expression of these genes in both male and female KO kidney, compared to WT littermate controls of the same sex. The magnitude of changes was comparable in animals of both sexes. Unpaired t-test was used for the comparison between WT and KO mice of same sexes. *: significance vs WT males; #: significance vs WT females. (G) n = 5 mice per group. (H, I) n = 3 to 8 mice per group. Scale bar 100 μm.

Pathological examination of kidney tissue from 8 weeks old mice confirmed the most prominent histopathological features of Alport syndrome in both female and male KO mice (Fig 2). Histological examination of hematoxylin/eosin (H&E) (Fig 2A–2C) and periodic acid-Schiff (PAS) (not shown) sections showed multifocal to diffused processes affecting the cortex and the medullary regions of the kidney. Many of the dilated tubules either contained eosinophilic material (casts) or showed degeneration/atrophy with short basophilic to coarsely vacuolated epithelial cells. Generalized involvement of the glomeruli with segmental to diffused obscuration of the glomerular structure (glomerular sclerosis) was also observed. The interstitium was multifocally thickened by few cells resembling fibroblasts and more rarely, inflammatory cells. H&E or PAS showed increased deposition of collagen at the corticomedullary junction or in the vicinity of sclerotic glomeruli. Sirius red stain showed diffused and exaggerated deposition of extracellular matrix (ECM) (Fig 2D–2F). Both sexes were affected equally by disease progression as indicated by nephropathy score and assessment of interstitial fibrosis (Fig 2G). F4/80 and α-smooth muscle actin (α-SMA) staining showed occurrence of fibrotic lesions characterized by infiltrates of F4/80 positive macrophages and α-SMA-positive myofibroblasts, in KO mice of both sexes (data not shown). Morphometric quantification confirmed a significant increase in macrophage and myofibroblast infiltrates in the KO kidney (Fig 2H). Male and female kidneys were similarly affected with 12- and 13-fold increases respectively, in α-SMA and 12-fold and 36-fold increases respectively, in F4/80 staining, as compared to age-matched WT mice. To correlate protein data with transcriptional expression the mRNA expression of Col3A1, F4/80 and Thy-1 genes were investigated by real-time PCR (Fig 2I). Previous studies have shown Col3A1 and Thy-1 (CD90) transcripts to be highly elevated in KO kidney [25]. Thy-1 has also been described to co-localize with α-SMA-positive myofibroblasts in several organs, including kidney, and represents a useful expression marker for myofibroblasts [26,27]. In agreement with previous studies, significant changes in Col3A1, F4/80 and Thy-1 gene expression are reported here, with 44-, 9-, and 18-fold increases in KO male kidney relative to wild type mice and similar extent of increases observed in KO females. The gene expression and protein composition data are consistent with the histopathological changes with no discernable differences in disease onset or progression between female and males KO mice. This observation and the absence of sex-associated weight differences with disease progression justified the use of only male mice henceforth in this study.

### Effect of macrophage-depletion on kidney function and fibrosis

**Fig 3:**
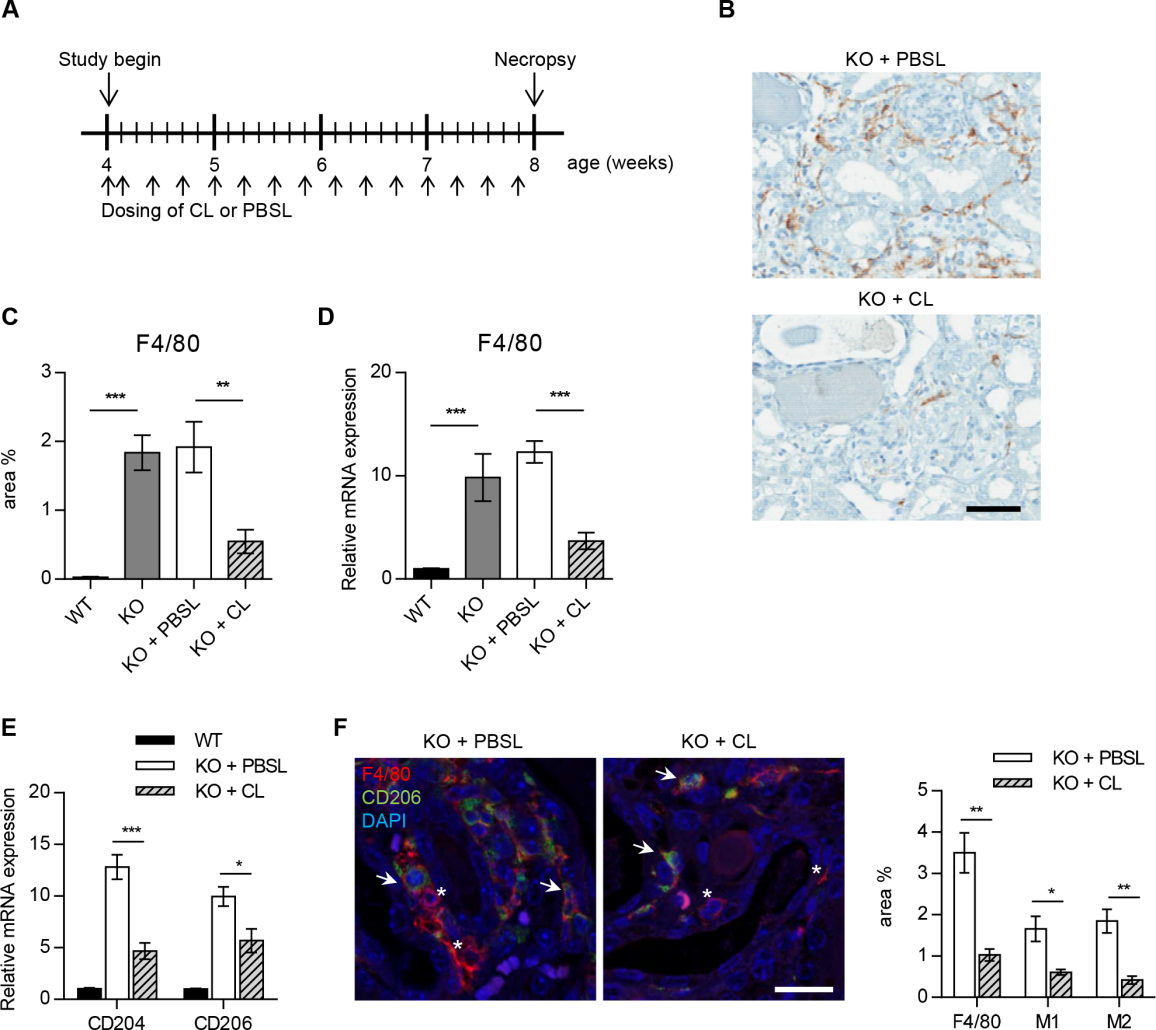
Clodronate liposome mediated macrophage depletion in Col4a3KO mice. (**A**) KO male mice were dosed intraperitonally with CL (KO+CL) or PBSL (KO+PBSL) as control. Animals were entered in the study at the age of 4 weeks and were continually dosed until the age of 8 weeks. Injections were repeated every second day, except for the first two doses, which were injected on consecutive days. (**B-D**) CL significantly reduced F4/80 positive macrophage infiltrates in KO+CL kidney as compared to KO+PBS or KO mice at the age of 8 weeks. (**B, C**) Representative images and quantitative assessment of F4/80 stained macrophages in kidney sections from KO+CL and KO+PBSL reveal marked reduction in macrophage infiltrates following CL dosing. Scale bar: 100 μm. (**D**) Real-time PCR analysis of F4/80 mRNA expression in KO+CL, KO+PBSL, KO, and WT littermates at the age of 8 weeks. Significant reduction in F4/80 mRNA expression following CL dosing was observed. PBSL did not affect endogenous F4/80 mRNA expression as shown by similar F4/80 mRNA expression in KO and KO+PBSL mice. (**E**) Real-time PCR analysis of CD204 and CD206 mRNA showed significant reduction in the expression of both genes in KO kidney upon CL treatment. (**F**) Representative images and quantitative assessment of F4/80 and CD206 stained kidney sections from KO+CL and KO+PBSL revealed marked reduction in M1 (F4/80^+^CD206^-^) and M2 (F4/80^+^CD206^+^) macrophages. Asterisk: F4/80^+^CD206^-^ M1 macrophages, arrow: F4/80^+^CD206^+^ M2 macrophages. Scale bar: 20 μm. Welch corrected ANOVA followed by posthoc pairwise t-test with a Bonferroni correction (**C,D,E**) and unpaired t-test (**F**) was used to evaluate differences between groups. (**C,D,E,F**) n = 5 mice per group. CL: clodronate liposomes; PBSL: PBS liposomes.

To investigate the contribution of macrophages to Alport disease, kidney macrophages were depleted in KO mice using clodronate liposomes (KO+CL) and compared to PBS liposome-treated mice (KO+PBSL), untreated Col4a3KO (KO), and wild-type (WT) littermate mice (Fig 3A). Treatment with CL and PBSL was initiated in 4-week mice since disease onset measured by proteinuria was not observed until 5 weeks of age [6]. Diffused infiltration of F4/80^+^ macrophages in KO or KO+PBSL kidneys was effectively inhibited through the administration of CL (Fig 3B). After 4 weeks of treatment with KO+CL, 70% macrophage depletion was observed as demonstrated by F4/80 protein and gene expression analyses (Fig 3C and 3D). Although, F4/80 is a well-known and widely-used marker of murine macrophages [28], it does not allow to discriminate between different macrophage populations. To evaluate the effect of CL on M1 and M2 macrophages, qRT-PCR was performed with M2 macrophage markers, CD204 (macrophage scavenger receptor 1) and CD206 (macrophage mannose receptor 1) [29–32]. Expression of both genes was downregulated following CL treatment in Col4a3KO mice kidney (Fig 3E). Furthermore, immunofluorescence staining showed reduction of M2 macrophages (F4/80^+^CD206^+^) as well as M1 macrophages (F4/80^+^CD206^-^) in KO+CL compared to KO+PBSL kidney (Fig 3F). Thus, confirming that CL depletes both M1 and M2 macrophages in Col4a3KO mice kidney.

**Fig 4:**
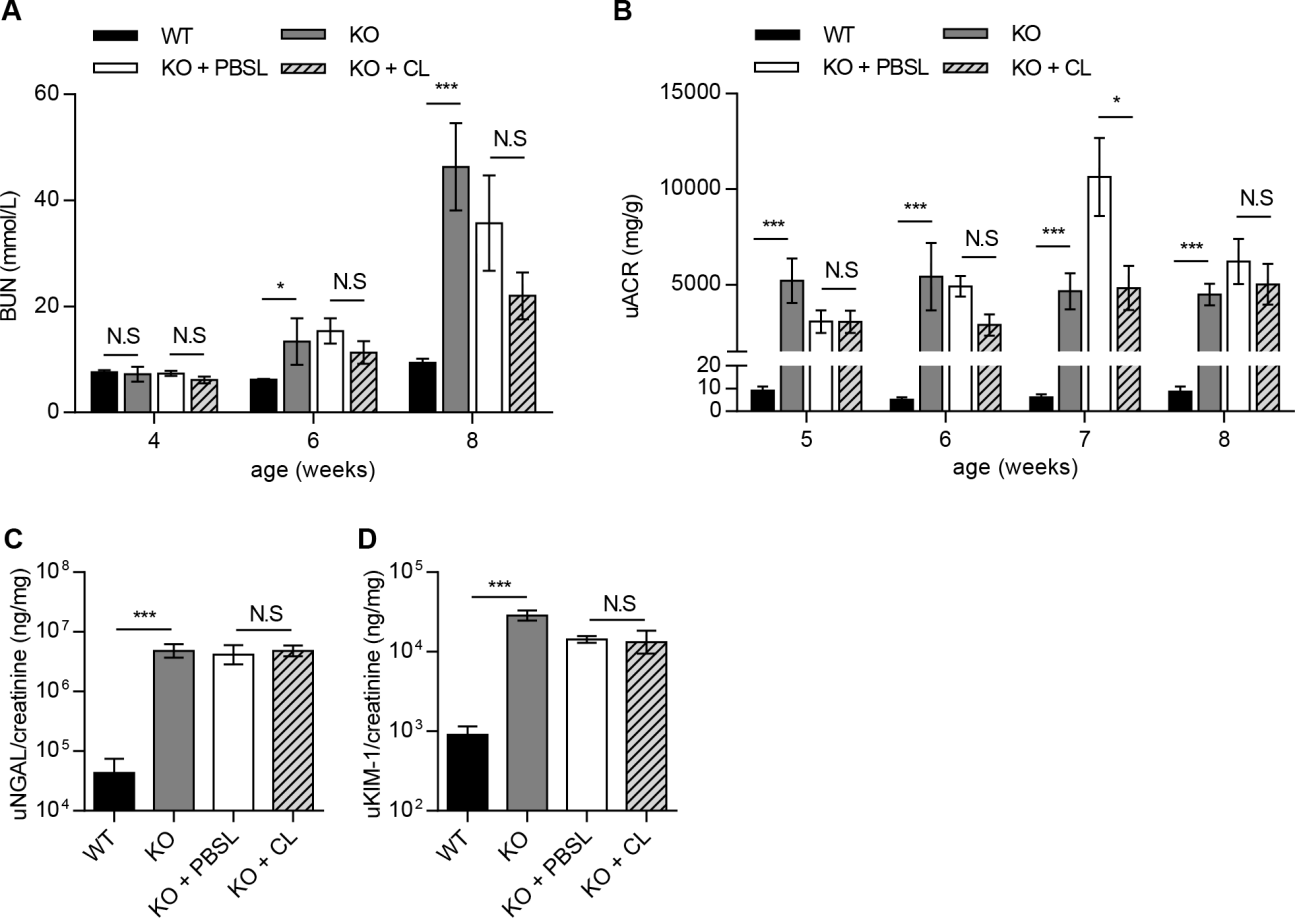
Macrophage depletion did not improve renal function in Col4a3KO mice. (**A-D**) CL treatment did not delay or slow down renal failure measured by serum BUN and urinary ACR, NGAL, and KIM-1. (**A, B**) Similar increase in BUN and urinary ACR was detected at all time points in KO+CL compared to KO+PBSL or KO mice. (**C, D**) KO+CL mice showed similar increase in urinary NGAL and KIM-1 as KO+PBSL or KO mice at the age of 8 weeks. Mixed model data analysis (**A, B**) and Welch corrected ANOVA followed by posthoc pairwise t-test with a Bonferroni correction (**C, D**) was used to evaluate differences between groups. (**A, B**) n = 5 to 9 mice per group. (**C, D**) n = 5 mice per group. CL: clodronate liposomes; PBSL: PBS liposomes.

**Fig 5:**
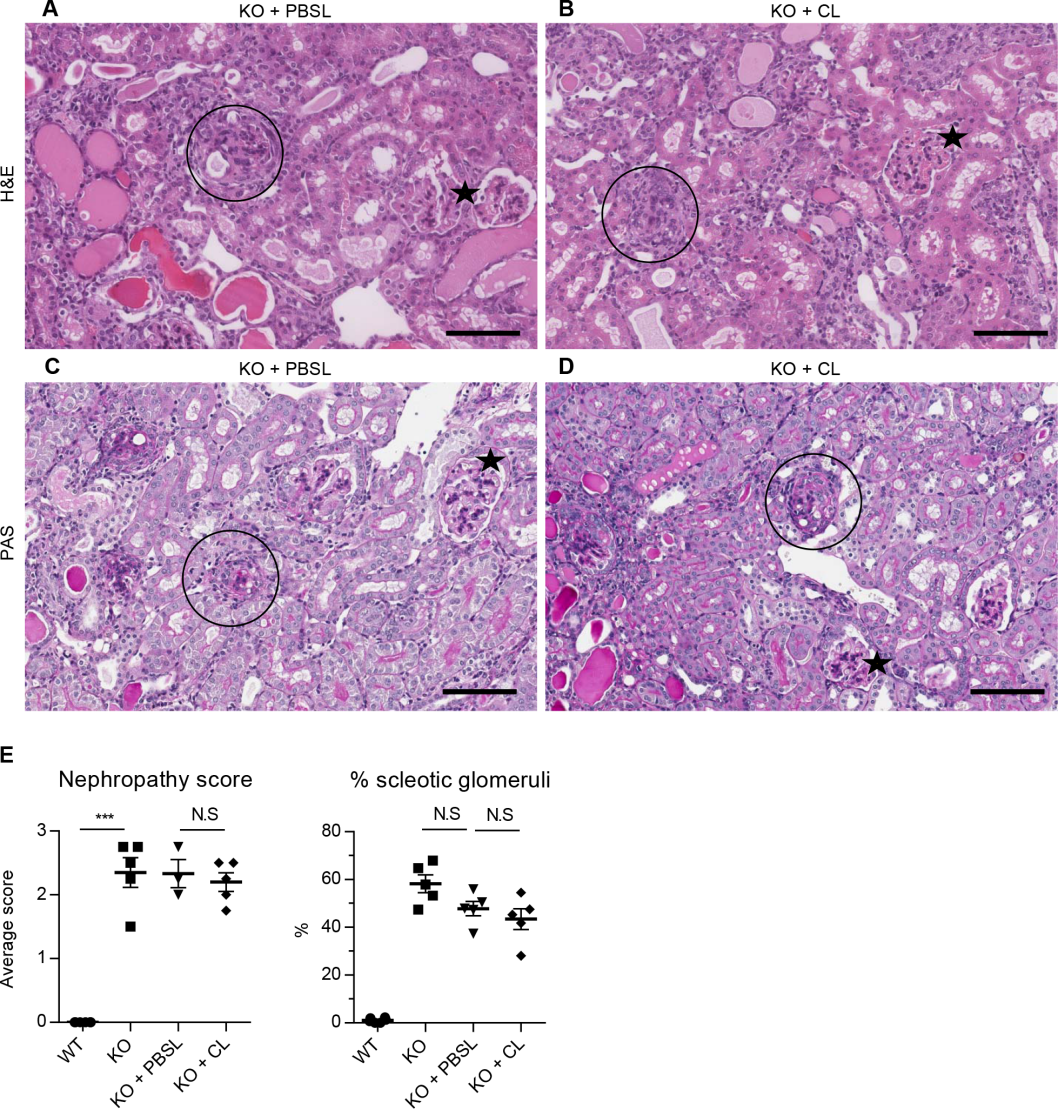
Macrophage depletion did not improve renal pathology in Col4a3KO mice. Kidney pathology was comparable between KO+PBSL and KO+CL. Panels **A** (H&E) and **C** (PAS) show tubular pathology (atrophy, degeneration/regeneration and eosinophilic casts) in KO+PBSL; panels **B** (H&E) and **D** (PAS) show comparable tubular pathology in KO+CL. Incidence of sclerotic glomeruli was comparable between KO+PBSL and KO+CL (circle = sclerotic glomeruli; star = normal-appearing glomeruli), with many glomeruli showing variable extent of changes from normal to sclerotic appearance. (**E**) Semi quantitative histologic assessment of nephropathy and sclerotic glomeruli revealed no significant differences between KO, KO+PBSL or KO+CL mice. Welch corrected ANOVA followed by posthoc pairwise t-test with a Bonferroni correction was used to evaluate differences between groups. (E) n = 3–5 mice per group. Scale bar 100 um. CL: clodronate liposomes; PBSL: PBS liposomes.

**Fig 6:**
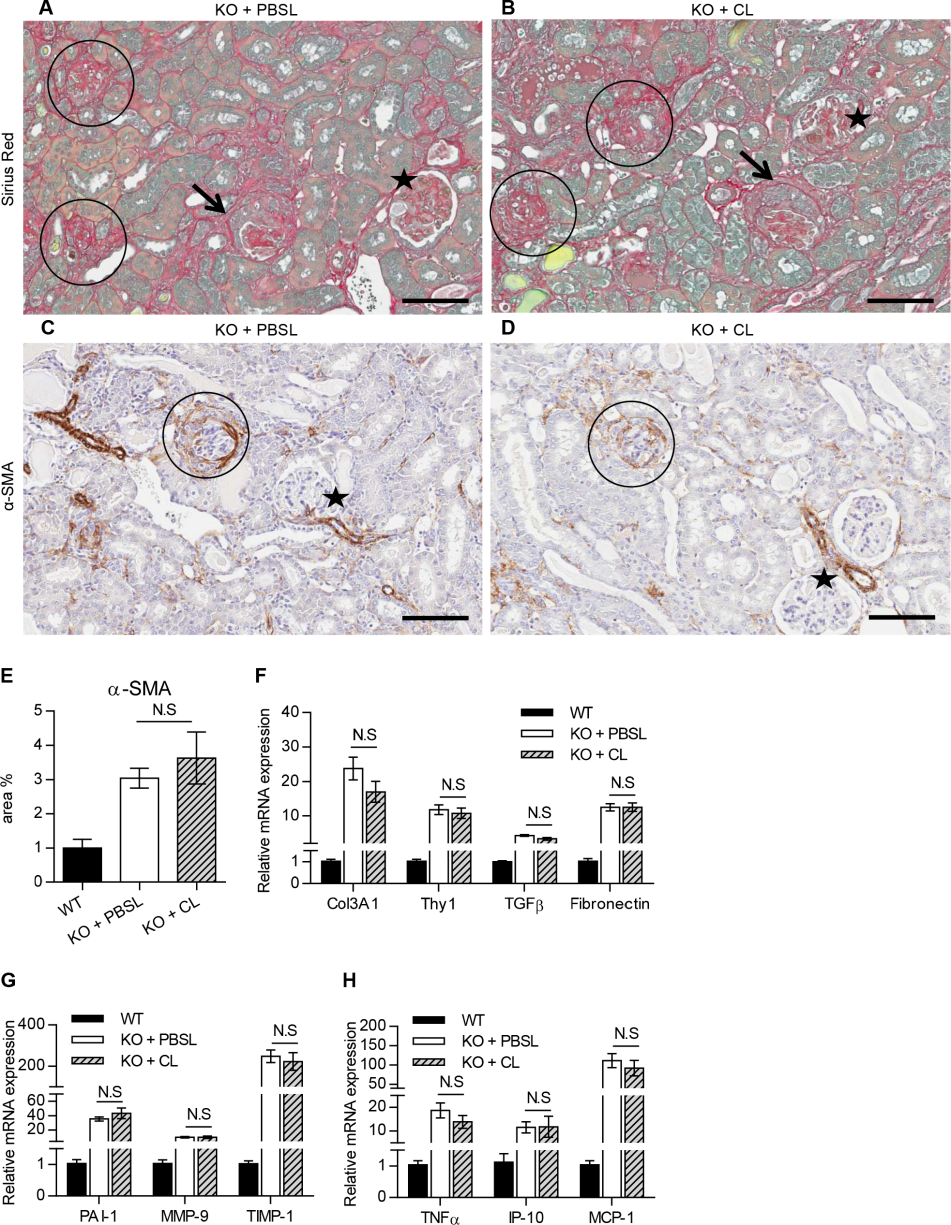
Macrophage depletion showed no effect on interstitial fibrosis and myofibroblast accumulation in Col4a3KO mice. **A** (Sirius red) and **C** (α-SMA) show increased deposition of Sirius red stained fibrotic tissue and α-SMA cells in the interstitium of KO+PBSL; **B** (Sirius red) and **D** (α-SMA) show an increased staining of the interstitium in KO+CL that was comparable to KO+PBSL, (circle: sclerotic glomeruli; star: normal-appearing glomeruli). Glomerular crescents (arrows) were also observed occasionally in glomeruli. (**E**) Morphometric analysis of α-SMA stained myofibroblasts confirmed no discernable effect of CL treatment on myofibroblasts deposition. (**F-H**) Expression of profibrotic genes (**F**), genes for ECM remodeling (**G**) and inflammation (**H**) is comparable in KO+PBSL and KO+CL mice. Welch corrected ANOVA followed by posthoc pairwise t-test with a Bonferroni correction was used to evaluate differences between groups. (E-H) n = 5 mice per group. Scale bar 100 um. CL: clodronate liposomes; PBSL: PBS liposomes.

The effect of macrophage depletion on the onset and severity of Alport disease was studied in Col4a3KO mice by assessing renal function and interstitial fibrosis. KO+CL mice progressed to renal failure with similar degree and severity as KO+PBSL-treated or KO mice (Fig 4). Kidney function was not improved as indicated by comparable levels of serum BUN and urine ACR, between the KO+CL- and KO+PBSL-treated mice within the 4–8 week time span (Fig 4A and 4B). A transient change in urinary ACR was measured only in 7-weeks old KO+PBSL-treated mice and could account for significantly lower levels in KO+CL-treated mice. No effect of macrophage depletion on the levels of NGAL and KIM-1 were observed in 8-week old mice (Fig 4C and 4D). Histological analyses did not reveal any differences in renal pathology, with all KO mice showing a similar extent in the severity and distribution of chronic renal pathology, regardless of the treatment received (Figs 5 and 6). The overall nephropathy score was not remarkably different following macrophage depletion (Fig 5E). A minimal decrease in glomerular sclerosis was observed in KO+CL mice when compared to KO mice, however there was no reduction when compared with the KO+PBSL mice (Fig 5E). Similarly, no reduction in α-SMA-positive cells was observed in KO+CL mice when compared to KO+PBSL mice (Fig 6C–6E). The elevated expression of profibrotic genes (Fig 6F) as wells as genes of ECM remodeling (Fig 6G) and inflammation (Fig 6H) did not differ in KO mice from different treatment groups. Although monocytes-macrophages are reported to be one of the cell types expressing TGF-β1 in the mouse Alport kidney [15], reduction of macrophages by 70% did not affect TGF-β1 expression, which appear to be increased 4-fold in KO mice as shown in this (Fig 6F) as well as in other studies [25]. This indicates that macrophages are likely not the major cellular source of TGF-β in the Col4a3KO kidney.

## Discussion

### Sex-independent progression of kidney disease in Col4a3KO mice

The human autosomal form of Alport syndrome has been shown to affect both males and females equally [12]. Similarly, mouse Col4a4 mutation causing Alport glomerulosclerosis, results in a similar progression of albuminuria in animals of both sexes [5]. However, little is known about sex differences in renal disease progression in Col4a3KO mice. This study demonstrates that the severity of disease onset and progression is not dependent upon sex of Col4a3KO mice. Thus, both female and male Col4a3KO mice are equally predictive of Alport syndrome and can be used to study pathogenic mechanisms and to evaluate experimental therapies.

NGAL and KIM-1 are produced by kidney in response to tubular epithelial damage [33,34]. Urinary KIM-1 and NGAL were systematically evaluated in Col4a3KO mice as markers of kidney injury along with BUN and urinary ACR, the standard measures of renal function. NGAL and KIM-1 were markedly elevated during early to late stage disease progression in Col4a3KO mice, supporting their role as markers of kidney damage. A similar pattern of increased NGAL excretion was also found in dogs with the X-linked form of Alport syndrome [35] suggesting a conserved pattern of NGAL expression in Alport nephropathy across multiple species. Further investigation would need to confirm the use of KIM-1 and NGAL as translational biomarkers of human autosomal recessive Alport disease.

### Macrophages depletion does not alleviate or prolong disease progression in kidney of Alport mice

To test the hypothesis that macrophage depletion would improve kidney function and renal pathology in Alport disease, Col4a3KO male mice were treated with CL. CL treatment, started prior to onset of disease as evidenced by clinical pathology and continued throughout the study, effectively reduced macrophage recruitment to the Alport kidney by ~70%. The extent of macrophage depletion in kidney obtained in Col4a3KO mice was similar to that reported in UUO where CL-mediated macrophage depletion prior to the UUO injury resulted in the amelioration of renal fibrosis [21].

However, the reduction of macrophages was not associated with improvement of histological or functional renal injury in Col4a3KO mice.

These data are in agreement with a previous study, which showed that significant inhibition of macrophage infiltration alone (via MCP-1/CCL2 blockage using anti-CCL2 spiegelmers) led to the reduction of glomerular and interstitial macrophages by 50% and 30%, respectively, but was not associated with improving renal pathology or prolonging the life span of Col4a3KO mice [18]. Although 70% macrophage depletion was achieved in the current study, any compensatory role by the remaining 30% macrophages in driving renal damage in Col4a3KO mice cannot be eliminated. Macrophage populations are not all the same, as shown by *in vitro* studies that differentiate between two major populations; M1 and M2, according to their response to specific cytokines. Both renoprotective and damaging effects have been attributed to M2 macrophages. Adoptive transfer of M2 macrophages has been shown to resolve inflammation and repair injury in many fibrosis models of kidney injury [36]. Similarly the ablation of macrophages during the M2 predominance is shown to slow kidney resolution in the reperfusion injury model [37]. On the contrary, conditional ablation of M2 macrophages defined as Ly6C^low^ has been shown to be antifibrotic in unilateral ureteral obstruction (UUO) model of kidney fibrosis [38]. Clodronate treatment can kill activated M2 macrophages [39] but the extent to which specific macrophage sub-populations are affected by clodronate in Alport syndrome has not been investigated in previous studies. We have shown that CL depletes both M1 and M2 macrophages in Col4a3KO mice kidney, and that the reduction in both macrophage populations does not slow down the progression of renal disease.

Partial depletion (25%) of interstitial macrophages via the antagonism of chemokine receptor 1 (CCR1), associated with a reduction of transendothelial migration of blood leukocytes, is reported to have a moderate effect on renal function as well as survival of Col4a3KO mice [17]. The results from our study indicate that macrophage depletion by clodronate treatment neither ameliorated, nor potentiated fibrosis in Col4a3KO mice. A possible explanation for the varied results regarding the impact of macrophage depletion in the progression of Alport disease is that a broad spectrum of leukocytes and not exclusively the monocyte/macrophage population are important for disease progression. In agreement with this, interstitial T-cell infiltrates are observed in renal biopsies of patients with Alport syndrome and are shown to inversely correlate with renal function of patients with Alport syndrome [40]. This hypothesis is also supported by observations from previous studies, in which Col4a3KO mice either crossed with RAG-1 deficient mice to lack mature lymphocytes or treated with a statin inhibitor to reduce both lymphocytes and macrophages alleviated Alport kidney pathology and prolonged survival. Specifically, Col4a3KO mice crossed with RAG-1-deficient mice showed reduced tubulointerstitial inflammation and fibrosis without improving the glomerular injury [41], while treatment with a statin inhibitor, had anti-fibrotic effect and prolonged the survival of Col4a3KO mice [42]. The data presented here strongly suggest that inhibition of macrophage infiltration alone is not sufficient to ameliorate progression of Alport syndrome in Col4a3KO mice and collectively with data from other studies [4,43] suggest that, targeting multiple immune cell populations will likely be more effective in checking kidney disease progression.
